# Engaging female community health volunteers in maternal health services and its satisfaction among village mothers in Hill and Mountain Regions, Nepal

**DOI:** 10.3934/publichealth.2020060

**Published:** 2020-10-09

**Authors:** Mina Lee

**Affiliations:** STOP-TB Partnership KOREA, Korean National Tuberculosis Association, Siem Reap, Cambodia

**Keywords:** female community health volunteer, maternal health service, maternal health, antenatal care, village mother, Nepal

## Abstract

**Background:**

Female Community Health Volunteers (FCHVs) are service providers and educators for maternal health at the village level (in the hill and Terai district) in Nepal. At present, there are insufficient data and little is understood about the maternal health service (MHS) of FCHVs from Nepali mothers' perspective.

**Methodology:**

The research was undertaken in three hill and mountain regions where there should be at least one FCHVs per ward, Thulo sirubari, Sano sirubari and Jalkeni in Chautara Sangachowkgadi in Nepal, during five days from 5 April to 9 April 2019. The study worked with a local partner organisation, Green Tara Nepal (GTN). The study took the form of a qualitative approach with a community-based snowball approach (seed-and-recruit approach), which consisted of interviews with six key informants and 11 village mothers who have a child aged under five.

**Results:**

The study found that all participated mothers recognised FCHVs and that it was easy to contact them within walking distance. They were happy with FCHV's existence and had a close relationship between them. They were all satisfied with MHS from FCHVs. In particular, accessibility of FCHVs, frequent home visits, monthly mothers' meetings, and regular ANC were the main points of satisfaction. Moreover, NGO intervention increased their satisfaction and contributed significantly to FCHV activities. However, the lack of meeting place and sustainable incentives are challenging to implement monthly mothers' meetings. Sufficient regular refresher training for FCHVs is also required to educate them better. Furthermore, the local government's unequally distributed budget for FCHVs programmes, small incentives, and benefits for FCHVs are challenges.

**Conclusion:**

This study offered various views with vivid memories into the satisfaction of FCHV's MHS among village mothers in three hill and mountain regions of Nepal. FCHVs are essential assets for MHS in rural communities. The FCHVs programme should be complemented by (1) supporting suitable meeting place of mother's meeting, (2) providing sustainable and sufficient budget for mother's meeting and FCHVs, (3) distributing the budget equally in each ward, (4) offering frequent FCHVs training to improve the MHS for village mothers.

## 1. Introduction

Nepal is one of South Asia's poorest countries and over 80 per cent of rural residents are living in hill and Terai districts [1]. The difficult geographic terrain of the diverse countryside isolates many people and thus deprives them of access to services. A study shows [2] that people living in mountain and hill region need to travel one to four hours to reach the closest health facility. Maternal Health (MH) is for women during pregnancy, childbirth and the postpartum period and maternal health care services are antenatal care (ANC), delivery care and postnatal care (PNC) services [3]. Maternal Health was the first concern of the National Health Policy in 1991 and it is the priority in the new National Health Policy of 2015. It has extended the Primary Health Care (PHC) system to the rural people, focusing on community-based actions providing the availability of free elemental health care services in the basic health facilities in the hill and Terai district [4].

The Female Community Health Volunteer (FCHV) programme was started by the Public Health Division of the Ministry of Health in 1988 to improve Primary Health Care (PHC) and to provide community-based outreach services focused on maternal and child health [5]. The FCHV is chosen by the mothers' group and they must be married and live in the ward where they are expected to provide health services [6]. Thus, village females frequently are open to discuss their pregnancies and giving birth with FCHVs [7]. FCHVs should receive 18 days of basic training regarding family planning, maternal, newborns, child health, and nutrition issues [8]. But also, they take in the refresh training every five years at the health post level and generally the village health worker of local health facility who is a supervisor meet them [9].

According to the report regarding FCHVs [6], FCHVs consist of two types: ward-based (hill and mountain regions) which is at least one FCHV per ward and population-based (the Terai), where the FCHV is allocated dependent on the number of residents. For example, one FCHV should have per 1,000 population in Terai and per 200 population in high mountains as currently 51,420 FCHVs have been working in each village [8]. Since the FCHV program began, maternal mortality rates (MMR) have decreased, reducing by fifty per cent from 1996 to 2006 [10]. There are many inventions that have been implemented through FCHVs distribute contraception (e.g., condoms and pills), vitamin A and iron tablets, Oral Rehydration Salts (ORS) packets and refer serious cases to health facilities and motivate and educate village members on healthy behaviour [8]. They are often credited for the improvement in MH in rural areas of Nepal [7],[11],[12].

In the rural villages of Nepal, there is competition among people between traditional healing practices and biomedicine. FCHVs contribute to the recognition of the importance of health facilities to local people [13]. According to a governmental report [10], FCHVs conduct monthly meetings, which mothers in the community attend, for discussing and sharing information such as antenatal care, delivery, postnatal care and family planning. Mothers' meetings led by FCHVs have contributed to the positive effects on maternal health. Community members have been increased the awareness of maternal health by FCHVs facilitating mothers' group [14],[15]. However, some mothers can find it difficult to attend due to insufficient time allocated for the meeting [16]. Some mothers have more time to discuss financial matters in their house instead of health issues during meetings [17]. FCHVs have a trusted position among community members and their contributions were recognised from home visiting [18]. Additionally, evidence showed that villagers have developed trust with FCHVs over time by their selfless volunteering of maternal services such as home visiting or door-to-door visits [19]. In addition, they notice the danger signs including the vaginal bleeding and heavy pain, severe headaches and trouble breathing in postpartum women and new-borns during postnatal home visits and they can advise the new mother how to take care of the baby [20].

The proximity to FCHVs enhances women who live in the communities they serve towards childbirth. Recent research shows that more than half of such volunteers arrived within 15 minutes after Nepali mothers gave birth [21]. FCHVs also work for disease prevention by providing supplements such as iron, and folic acid tablets to pregnant women [17]. Evidence shows that the prevalence of anaemia during pregnancy reduced from 67 per cent to 35 per cent from 1998 to 2011 [23].

However, villagers still doubt the provided services from the FCHVs. It had previously been observed that nearly 90 per cent of female community health volunteers did not visit each home to check baby or children who were sick in their villages [24]. As shown by this study, it also stated that 67 per cent of Nepali mothers did not request FCHV's service due to poor their service. As reported by the Nepal Demographic and Health Survey (DHS), only 3 per cent of village mothers visited FCHVs when their children fall ill [25]. Some findings also indicate that more than half the pregnant women and mothers who received underused services in pregnancy, delivery, and postpartum newborn care insisted FCHVs provided inadequate care with insufficient information and a lack of interpersonal manners [26]. FCHV's lack of friendly manners, skill and knowledge have been raised, respectively [10],[24]. Other studies have identified that some mothers experienced poor attitudes and ignorance from FCHVs [27]. The study [6] also found that only 62 per cent of FCHVs were literate, but literacy levels did not have an impact on the quality of services provided by them. On the other hand, it has been counter-argued that only some FCHVs did have the knowledge and skills for the correct use of spring scales, which have led to overestimation of infant weight [21]. Most villagers in rural areas are likely to visit traditional healers first when they are sick [28].

At present, there are insufficient data and little is understood regarding how the MHS' task of FCHVs are viewed and experienced by Nepali mothers [29]. Thus, this research aimed to explore how to engage FCHV's maternal health services among Nepali mothers. And it was for understanding how they feel the satisfaction of FCHV's MHS and for investigating their thought and opinion of them. In addition, it was the objective to find out the possible challenges to the FCHV's MHS among them. This paper examines the case of the FCHV programmes in Nepal, an extensive community-based intervention for the management of MH in rural areas. The qualitative research design was established by interviews with Nepali mothers and key informants such as FCHVs themselves, health workers and a non-governmental organisation (NGO) director. Further, the study is based on the perspective of village mothers who live in hill districts.

## 2. Methodology

The research was undertaken in the three hill and mountain regions where there should be at least one FCHVs be per ward, Thulo sirubari, Sano sirubari and Jalkeni in Chautara Sangachowkgadi Municipality in Nepal, during five days from 5 April to 9 April 2019. This study was for the overseas field class of International Development Masters programmes of the University of Sheffield. This course was for providing postgraduate students direct experiences of the research in Nepal. The study worked with a local partner organisation, Green Tara Nepal (GTN), which have cooperated for several years for this course. Practically, it was necessary to work in groups, which are a common research topic. A team of four postgraduate students, one local guide, and one Nepali interpreter consisted. The maternal health topic and common methodology were pursued together. However, we had different research focused on that topic. The study took the form of action research as a qualitative approach with a community-based snowball approach (seed-and-recruit approach), which consisted of interviews with six key informants and 11 village mothers. Village mothers who have a child under five years were consisted of getting vivid experiences from FCHVs on MHS. Most of them had given birth recently.

### 2.1. Participant selection and data collection

Key informants were interviewed to address the research questions and identify the perception of the role of FCHVs. Two formal interviews (one individual interview and one focus group interview) were conducted with key informants. Key informants included one FCHV, two midwives, and one GTN director as individual interviews, and one focus group interview was held with two FCHVs and one midwife. They were recruited by GTN staff and local field guides. The participants were consisted of married women who have a child aged under five in Thulo sirubari, Sano sirubari and Jalkeni villages. They were recruited through snowball sampling supported by the help of a local field guide and GTN staff.

Opened ended and semi-structured interviews were used for the study among key informants and village women. This method was selected because interviews enable them to provide much insight into the personal experience [30]. It gives the interview direction by allowing the flexibility of individual responses [31]. Key informants were focused on collecting various aspects of FCHV's work, such as their role and responsibility, relationship, improvement and challenges of FCHV's MHS. It was helpful to address the research questions for interviewing with village mothers. In-depth interviews were conducted with 11 village mothers. Questions including the experience, relationship, satisfaction, improvement, and challenges of FCHV's MHS were asked, but they were edited to investigate new findings. Individuals and focus group discussions were held in private areas. All interviewed were conducted face-to-face in Nepali, recorded, and translated into English. The duration of the interviews was approximately 20 minutes. Field notes were taken during or immediately after the interviews. This was because field notes enable the development of essential frameworks for the transcription of recording data as well as acting as reminders of the situational factors during analysis [32].

### 2.2. Data analysis

All interviews ware translated into English from Nepali as supported by a Nepali interpreter and transcribed. Data analysis was conducted after all data was collected. The data collection was undertaken in Nepal and data was analysed in the UK. Data were analysed using thematic analysis. Thematic analysis enables to identify and analyse the themes within the context of the data collected [33]. The transcription was read and re-read for familiarity and was highlighted the phrases and sentences. These were formalised into codes by taking different colours. Codes were categorised and generated into themes. Main themes were based on four concepts such as relationship, satisfaction, improvement and challenges of FCHVs on MHS. This found key concepts and recurring ideas that consistently occurred during the data collection process and formed the basis of the finding reported.

### 2.3. Ethics consideration

The study procedure was approved by the University of Sheffield Geography Ethics Committee. Before starting the interviews, all participants were orally presented with the research aims and approach, and oral consent was requested of them in the research. In addition, they were informed that they could withdraw without giving a reason if they did not want to continue. If the participant agreed, the interview was recorded using a digital tape recorder. In this case, verbal consent was audio-recorded again as a further confirmation. All interviews were carried out by a Nepali interpreter and translated into English. All interviews were held in a private space, which was away from family members and crowds of people. To collect a realistic data set, questions remained general throughout the interviewing process and were not adapted in response to information gained in previous interviews. Moreover, transcripts were anonymised, audio recordings were password-protected, and data were saved in a protected file store of the University of Sheffield.

## 3. Results

Overall, 11 mothers were interviewed. Six key informant interviews were undertaken such as three FCHVs, two midwives and one NGO director (see Table 1). They are all local people. None of the approached individuals refused to participate in the research or dropped out during the interview. The finding was explored by the themes such as the relationship, satisfaction, improvement and challenges to FCHVs on MHS from the data and from experiences of interviewees.

**Table 1.** Characteristics of interviewed participants.

Respondents	Place	Gender	The number of children and age	Position Held	Remark
Village Mother (VM)
VM2	Thulo sirubari	F	1/11months	Household	
VM3	Thulo sirubari	F	2/9 years and 2 years	Household	
VM4	Thulo sirubari	F	1/10months	Household	
VM6	Thulo sirubari	F	3/10 years, 6 years and 9 months	Household	
VM7	Thulo sirubari	F	2/7 years and 1.5 years	Self-employed	
VM8	Sano sirubari	F	2/7 years and 1 year	Household	
VM9	Sano sirubari	F	2/7 years and 2 years	Self-employed	
VM10	Sano sirubari	F	2/5years and 6months	Household	
VM11	Sano sirubari	F	2/3years and 2years	Household	
VM12	Jalkeni	F	2/8years and 4 years	Household	
VM13	Jalkeni	F	3/9years and 3 years and 1.5 years	Household	
Key Informant
Midwife 1	Thulo sirubari	F		Midwife	
FCHV5	Thulo sirubari	F		FCHV	
Midwife14	Jalkeni	F		Midwife	Focus group
FCHV15	Jalkeni	F		FCHV	
FCHV16	Jalkeni	F			
GNT director 17	Jalkeni	M		GTN director	

### 3.1. Close relationship

Most interviewed village mothers often answered “good” or “close” regarding the relationship between villagers and FCHVs. It was found that some village mothers used to openly talk with the FCHVs even about their personal lives. A pregnant female highlighted: *“I have a good relationship with the FCHV. She is very friendly. I used to talk with her about my concern or family. She gave me many advices. She frequently visited me to check my condition and inform me related to the baby.”* [VM 2].

Moreover, during the focus group discussion with FCHVs, they insisted that the relationship among community members had been improved more closely. One of them explained it: *“Close relationship has been increased a lot. Now women have started to talk about their family planning and methodology. Because it is closed, they want to talk about it more. Earlier, they did not talk about some sensitive issues like them and they felt very shy. Now they come to me and ask about temporary contraception, like ‘what should I use... I want...’”* [FCHV 5].

The above quote demonstrates that village women and FCHVs have started to communicate about the delicate issue. Talking about the contraceptive method or family planning with FCHVs, village women tend to feel “closer” to FCHVs and, thus, more able to talk about it. Therefore, it was affirmed again that they have a close relationship.

### 3.2. Satisfaction of FCHVs on MHS

Most interviewed mothers were satisfied with the FCHVs' skills and services. Accessibility of FCHVs, frequent visiting home, monthly mothers' meetings and antenatal check-up are common answers about what they were satisfied with. In this part, the finding in relation to the satisfaction of village mothers will be discussed.

#### 3.2.1. Accessibility of FCHVs

The villages interviewed each have FCHVs. All participants know them and their role and responsibilities. Most village mothers said that FCHVs are accessible and ready to help when their children were sick and also, they could approach them at any time. They appreciated the presence of FCHVs in their community, as commented by one respondent: *“I am happy that she is in our ward and helps us”* [VM 9]. The GTN director highlighted that the role of FCHVs recognises values for the community, commenting, *“Nearly 90 per cent of women in Nepal know FCHVs and local people consult with them on issues like ‘I have diarrhoea, what do I have to do?’ and they are happy with the existence of FCHVs and they appreciate their contribution to the local community.”* [GTN director 17].

#### 3.2.2. Frequent visiting home

It was analysed that frequent visiting home is a factor that creates satisfaction with FCHV's MHS among interviewed village mothers. It was identified that visiting home is one of the main roles of FCHVS in providing the consultation. As one mother stated: *“She often came to visit my home to see my child and asked what is happening with my child's health and my health.”* [VM 11].

Moreover, they used to refer village mothers to go to health facilities (e.g., hospital and health post) for the MHS utilisation. *“She often came to my home, saying no no no. Take them to the hospital or suggest my family members take to the hospital.”* [VM 9]. By this effort, it was revealed that village women are likely to go to health post or hospital, *“FCHV reminded me to go the health post whenever my child is sick. I haven't visited the traditional healer.”* [VM 7]. From this quote, it can be assumed that rural communities do not recognise traditional healers as a popular source of health treatments. However, the study found that women living in Jalkeni do not necessarily have to request help from FCHVs because they already knew the awareness of health facilities well. A group interview with FCHVs commented: *“Before I taught them like you should go to the hospital to delivery or check-up ..., but now women visit the hospital themselves”* [Focus group, FCHV 15]. It leads to deep thinking that village women could gradually no longer seek FCHVs services. Therefore, the role of FCHVs could be less.

#### 3.2.3. Monthly mothers' meeting

A monthly meeting with FCHVs is the major drive in achieving improved maternal health knowledge and giving satisfactory service. They are used to educate on birth preparedness, nutrition, childcare, vaccination, cleanness and family planning. Most participated mothers mentioned the monthly mothers' meeting as “useful”, “effective”, and “satisfied”. Notably, the monthly meeting provides not only health education, but also FCHVs to ask them to bring their baby to do a medical check-up. One mother mentioned: *“Once we attended a monthly meeting, she [FCHV] asked us to please bring our child to the measurement and she tell us if the child is healthy or not.”* [VM 7].

One of the surprising findings of this research was that some women are more satisfied with monthly meetings than with home visiting. *“When I go to such a meeting, I can discuss the child health as well as the FCHV used to take my child's measurement and tell me if he is growing healthily or not. But that doesn't happen in the home.”* [VM 9].

On the other hand, some mothers hoped that the number of mothers' meetings could be increased. One mother said: *“The monthly meeting happens once a month. If it happens more, twice a month, it will be more beneficial for women like me.”* [VM 2].

#### 3.2.4. Antenatal check-up

According to the National Female Community Health Volunteer Program Strategy [10], it stated that primary role of FCHVs is safe maternity, child health, family planning and other community-based care to promote the health behaviour of mothers and community people. This study identified that a major role of FCHVs find new pregnant women in their ward and report them to the health post. Thus, pregnant women can readily approach the maternal health services for support from FCHVs, who will provide information, iron tablets and refer them for at least four times antenatal check-ups (ANC). pregnant females have been managed the prenatal care well by FCHVs as seen quote below. *“Finding the pregnant women is the main responsibility. If a couple recently got married in my village, I just visit them to teach family planning and ask them to let me know if they have any symptoms of pregnancy. Or if she says that she is pregnant, then I have to write her name and her last period data and how her feeling. And then I have to report it to the health post. So pregnant women can be registered in the health post”* [Focus group, FCHV 15].

This highlighted the emphasis on the effort of FCHVs towards prenatal check-up: *“The FCHV said that I should go for an antenatal check-up at the health post”* [VM 9]. Analysing this expression, and from other similar answers of mothers, it apparent that the *“FCHV encouraged me to go to the health post to have a check-up while I was pregnant”* [VM 11]. Even after mothers gave birth, it was found that they were satisfied with the FCHV visiting their home frequently to check on their baby. A quote from a mother in Sano sirubari village in given below: *“I was satisfied with the FCHV. Once a child is born, they visit very frequently checking what immunisation the child needs to give a vaccination.”* [VM 10].

Additionally, it was found that a Nepali mother was satisfied with prenatal care of FCHV during her pregnancy period: *“When I was pregnant, the FCHV gave me iron and calcium tablets and she visited my house to check my condition. I was satisfied and I was able to concern about my pregnancy more.”* [VM 7].

### 3.3. NGO intervention

NGO intervention enables FCHVs to maximise the satisfaction among village members. It was found that Medic Mobile, which is an NGO, offers a mobile phone and application to FCHVs for follow-up with ANC. According to a recent study, Medic Mobile has increased the frequency of contact with mothers and newborns, and increased regular home visiting provided by FCHVs [34]. One midwife mentioned that this application provided a proper service for both FCHVs and village mothers. *“Once FCHVs register some pregnant women in the Medic application, during ANC care time, such as every 4, 6, 8 and 9 months, automatic messages will be sent to them. It is very effective to monitor and more pregnant women are enabled to visit here for check-up compared to the past. They are quite content with the referral services from FCHVs.”* [Midwife 1].

Moreover, it is observed that village women are enabled to get ANC care at a suitable time. One FCHV highlighted this as compared with the past. *“Before, many pregnant women did not come for antenatal check-ups in time. This application is going to help us by sending the automatic reminders. The rate of women's regular ANC has increased.”* [FCHV 5].

In addition, it is observed that some incentive, such as food or light snack, by the supported NGO has enabled to have high attendances of the monthly mothers' meeting. Therefore, it clearly showed that village mothers were able to improve their maternal health knowledge as well as boosting greater satisfaction with the FCHV's service. A participated FCHV pointed out as below: *“A working health organisation named Suarhara used to provide food to the women who attended monthly meetings held by FCHVs. When they provided lunch in that meeting, the number of participants was very high and they learnt various health educations related to their baby.”* [Focus group, FCHV 16].

Another participated mother also highlighted that FCHVs used to educate how to feed with offered incentive, which led to improve satisfaction. *“They [FCHVs] have given some planta and flowers to prepare the food for my baby. Flower is cooked with hot water. It is made for the baby. It is very useful and I am very satisfied with this service.”* [VM 13].

Overall, the finding makes it evident that mobile application and some incentive from the NGO can have positive effects on managing regular antenatal care check-ups and improving maternal health knowledge, which has led to boost the satisfaction among village women. However, village mothers in study areas could significantly be challenging the sustainable development of maternal health with FCHVs if the NGO project is completed.

### 3.4. Challenges

Interviewed participants identified some main challenges that impact on FCHV's service to meet satisfaction.

#### 3.4.1. Refresher training

Some village women complained that FCHVs need to have more wider knowledge. One mother explained: *“Among the FCHVs, some of them are clever and educated, but some of them lack education”* [VM 4]. It was found that some FCHVs have taught based on personal experience or from other experience of trained FCHVs. Some participated village mothers criticised their education as like “the topic is always same” and “she depended on her experience”. One FCHV also highlighted that regular refresher training is essential to remind them so that they can update knowledge. She said: *“We do not always remember what we have learnt. We should be trained regularly to remind us. This could help us perform better in our community.”* [FCHV 5].

On the other hand, it was found that new FCHVs have not still been given proper training by the government, as one interviewed FCHV said *“they are still waiting the training”*. Hence, the government should plan frequent refresher training and new training between FCHVs and new FCHVs to provide more informative and effective education for villagers.

#### 3.4.2. Lack of places & financial support

Some villagers argued that they do not have a proper place to gather for the monthly mothers' meeting and this is a particular challenge. One of them complained: *“we just put out some mattresses and our children move around during the meeting”* [VM 3]. Analysing this expression, and from other similar answers, such as *“the mother's meeting is held on the roof of the health post. This place is open. It should have a proper place”* [VM 13].

Moreover, notably lack of benefits, such as discount on fees for utility fee or school fee or small incentives, have been raised by FCHVs as their primary challenge. This is likely to cause a lack of maternal health services quality for village mothers. A FCHV pointed out: *“We [FCHVs] aren't ready to help without any incentives. Government provides us 1000-200 rupees per months. It is a very small incentive. We do some needs. If governments can help with some more benefits, it will encourage us to do services for them [village members].”* [FCHV 5].

Remarkably, it was revealed that the budget for the FCHV programme from the local government has not been distributed equally to each ward. This has meant that FCHVs in each ward are not able to get the same incentive equally; they could not even provide the snacks for the mothers' meeting. A FCHV highlighted this as below: *“As you know, that there are nine wards, and some are not given a fee and some are given enough budget for providing snacks and incentive. We requested the local government that each ward should have the same budget. If there is a proper budget for any problems in handling maternal health services, it could improve its service.”* [Focus group, FCHV 15].

The above statement makes it evident that unequal budget has been distributed in each ward and a proper budget is essential to improve maternal health services.

Furthermore, a sustainable budget for the monthly meeting could be challenging. As mentioned above, the monthly meeting by FCHVs has a positive effect on education in MHS for village mothers. However, it was found that women are less likely to attend a monthly meeting if they are not offered food or snacks. The current situation was explained by a FCHV as below: *“Food kind of incentive encourages them [Women] to create more enthusiasm and more attendance. Their enthusiasm is a little bit less than the past, because food was not provided in the meeting. Even though the local government provided a small budget each month for a monthly meeting, it is not enough for them to provide individual food.”* [FCHV 5].

The above statement makes it evident that a sustainable budget for monthly meetings is needed to create village mothers' enthusiasm.

## 4. Discussion

This study aimed to investigate the engagement and satisfaction of FCHVs on MHS among village mothers in hill and mountain regions. All interviewed Nepali mothers were satisfied with FCHV's MHS. Accessibility of FCHVs, frequent home visiting, monthly mothers' meetings and ANC check-ups are common satisfied factors from FCHV's MHS. All participated Nepali mothers were happy that FCHVs are in their ward and they recognised their role and responsibility. This research found that FCHVs are accessible and ready to help mothers in the villages and could be approached at any time [21]. The finding is clear that frequent home visiting is a major satisfaction factor among village mothers [18],[19]. It encourages them to learn child care and to use maternal services in health facilities, such as hospitals and health posts [13]. Notwithstanding, as village women have increased their awareness of the health facilities, the role of FCHVs which was used to promote them is decreased. The government must consider reorganising their role and responsibility.

Contrary to the literature review [28], it was analysed that traditional healers are not recognised as the primary health source in interviewed areas. Villagers are likely to visit the health post instead of visiting traditional healers. Moreover, it was clear that they have a very close relationship [18],[19] with FCHVs in sharing their concerns and sensitive topics (e.g., contraception methods and family planning) without any hesitation. However, this finding adds weight to the wider literature arguments that some village mothers experienced poor attitudes and lack of interpersonal matters [10],[24],[26],[27].

Furthermore, as prior studies [10],[14],[15] stated, it was clear that the monthly meeting of FCHVs leads to improved knowledge of maternal health among village mothers. Also, it was identified that FCHVs used to visit the pregnant woman's home to inform them of ANC education and check their condition [20]. The statement adds weight to dispersing the debates in the literature, arguing lake of care during pregnancy or childbirth [26]. Notably, NGO intervention has driven to create more satisfaction, and providing mobile phones and applications and some incentives (e.g., food and snacks) has contributed FCHVs to manage effectively in educating village mothers on MHS. In particular, I found that mobile applications help FCHVs readily to check the timing of ANC check-ups for pregnant women. Thus, new technology such like this has been used among FCHVs and it ensures village women are well-treated, taken care of, and properly guided towards health check-ups during their pregnancy. In addition, adequate incentives for monthly mothers' meetings were expected to make women more enthusiastic about learning and attending [35]. However, this study found significant challenges in FCHV's MHS. Most village mothers complained about the places they used to learn, such as distracted and open spaces, at the monthly meeting.

Moreover, it was clear that the knowledge of FCHVs still concerns them [10],[21],[24]. Furthermore, FCHVs felt that frequent training is essential to improve the MHS. A sustainable incentive such as food or snacks is required as, currently, there are fewer numbers of attended village mothers and a lack of enthusiasm for meetings being organised in hill regions. Additionally, it was found that improper distributed budgets for FCHV programmes from local government to each ward are challenging. Small incentives or benefits for FCHVs are still present [36].

This paper aimed to contribute to a deeper understanding of the engagement of FCHV's MHS and how village mothers have felt satisfied with their services. This paper was conducted with Nepali mothers how mostly had given birth recently. Thus, it was able to get lively information and experience from them. If the current challenges which have been found can be solved, it is believed that FCHVs could contribute towards better MHS in hill and mountain communities. Also, Nepali mothers could feel more satisfaction and improve maternal health.

## 5. Conclusions

This scoping study provided various views into the satisfaction of MHS from FCHVs in three hill and mountain regions of Nepal. All participated mothers recognised FCHVs and contacted each other often within walking distance. They were happy with FCHV's existence and had a close relationship with them. They were all satisfied with MHS from FCHVs. In particular, accessibility of FCHVs, frequent home visits, monthly mothers' meetings, and regular ANC are the main points what satisfaction.

Moreover, NGO intervention has increased their satisfaction and contributed significantly to FCHV activities. Thus, FCHVs are essential assets for MHS in rural communities. However, the lack of meeting places and sustainable incentives are challenging to implement monthly mothers' meeting. Sufficient regular refresher training for FCHVs is also required to educate them better. Furthermore, unequal distributed budget from the local government for FCHVs programmes, small incentives, and benefits for FCHVs are also challenges. Thus, these are crucial issues to consider to improve the function of FCHVs in maternal health services.
